# Role of *Toxoplasma gondii* p24δ in Regulating the Transition from Tachyzoite to Bradyzoite Development

**DOI:** 10.3390/ijms26073331

**Published:** 2025-04-03

**Authors:** Zifu Zhu, Zhu Ying, Yanqun Pei, Zhili Shan, Jing Peng, Ming Sun, Qun Liu, Jing Liu

**Affiliations:** National Key Laboratory of Veterinary Public Health Security, Key Laboratory of Animal Epidemiology of Ministry of Agriculture and Rural Affairs, National Animal Protozoa Laboratory, College of Veterinary Medicine, China Agricultural University, Beijing 100193, China; s20193050765@cau.edu.cn (Z.Z.); yzyingzhu@163.com (Z.Y.); 18349325943@163.com (Y.P.); 18801286020@163.com (Z.S.); yyqxpengjing@163.com (J.P.); bs20223050478@cau.edu.cn (M.S.); qunliu@cau.edu.cn (Q.L.)

**Keywords:** *T. gondii*, Tgp24δ protein, bradyzoite-specific genes, bradyzoite, differentiation

## Abstract

*Toxoplasma gondii* is an obligate intracellular parasite capable of infecting warm-blooded vertebrates, including humans. In its intermediate hosts, *T. gondii* can transition between two life stages: the rapidly replicating tachyzoite and the quiescent bradyzoite. In *Saccharomyces cerevisiae*, the p24 protein acts as a cargo receptor, cycling between the ER and Golgi in the early secretory pathway to recruit cargo proteins into nascent vesicles. However, the function of p24 in *T. gondii* remains undefined. In this study, we identified four p24 proteins in *T. gondii*, with Tgp24δ specifically localizing to the ER–Golgi system. Loss of p24δ in a type Ι strain (RH*Δku80*) significantly reduced proliferation and virulence in mice. Transcriptome and proteomic analyses showed that Tg*Δp24δ* tachyzoites expressed high levels of bradyzoite-specific genes, including *bag1*, *ldh2*, and *bpk1*, under standard culture conditions. Additional data indicate that Tg*Δp24δ* tachyzoites can differentiate and form bradyzoites in vitro. This suggests that Tgp24δ is important for the parasite’s growth.

## 1. Introduction

*Toxoplasma gondii* is an obligate intracellular protozoan capable of infecting the nucleated cells of almost all warm-blooded animals, including humans [1]. The primary routes of *T. gondii* infection in humans include the ingestion of either undercooked meat containing tissue cysts or contaminated vegetables harboring oocysts [2]. Once ingested, the parasites transform into tachyzoites, which spread throughout the body and ultimately form tissue cysts. This can result in severe central nervous system lesions in immunocompromised patients or lead to abortion and stillbirth in pregnant women and animals [3]. Additionally, when the immune system is compromised, latent infections can reactivate, with bradyzoites converting back to tachyzoites, potentially leading to encephalitis or other diseases [4]. Therefore, understanding the molecular mechanisms of parasite differentiation is crucial to elucidating the pathogenesis of *T. gondii*.

Previous studies have identified that several physicochemical stressors can induce bradyzoite differentiation, including alterations in pH (8.2), heat shock (43 °C), nutrient starvation (pyrimidine and arginine starvation), cholesterol deprivation, and exogenous nitric oxide [5,6,7,8,9]. A common characteristic of these stressors is their ability to inhibit tachyzoite replication [10,11]. In addition to these stressors, it has been reported that the deletion of some key genes in *T. gondii* can slow down the proliferation rate of the parasite and trigger the transition from tachyzoite to bradyzoite. For example, genetic ablation of TgPKAc3 in a type I strain (RH*Δku80Δhxgprt*) led to slower growth compared to the parental strain and a higher basal rate of tachyzoite-to-bradyzoite differentiation [12]. In the double-knockout strain Δmca1Δmca2 in RH*Δku80*, growth was significantly reduced and tachyzoites differentiated more readily into bradyzoites in vitro [13]. Despite many studies on cyst formation, the mechanisms underlying stage interconversion remain unclear.

In eukaryotic cells, p24 family proteins present on COPI and COPII vesicles are categorized into four subfamilies: p24α, p24β, p24γ, and p24δ [14]. Structurally, p24 proteins are small (20–25 kDa) type I membrane proteins which typically consist of luminal Golgi dynamics (GOLD), a single transmembrane domain, and a cytoplasmic tail harboring COPI- and COPII-binding motifs [15]. They are generally considered cargo receptors that play an essential role in efficient protein sorting [16]. In *Saccharomyces cerevisiae*, deletion of p24β (Emp24p) or p24δ (Erv25p) resulted in delayed transport of the glycosyl phosphatidyl inositol anchoring protein (GPI-AP) Gas1p [17,18,19]. In *Arabidopsis*, p24 proteins are associated with ER export and trafficking of GPI-APs to the plasma membrane [20]. In mammalian cells, TMED10 promotes ER export of insulin-like growth factor 2 (IGF2) via recognizing an export signal on IGF2 [21]. Overall, the p24 family proteins are widely conserved across eukaryotes and play a crucial role in maintaining the flow of proteins through the secretory pathway in various organisms.

However, the function of the p24 protein in *T. gondii* and its role in tachyzoite-to-bradyzoite differentiation remain unknown. To gain a better understanding of the diverse functions of p24 protein in *T. gondii*, we first identified members of the p24 family proteins in the *Toxoplasma* genome. Subsequently, we constructed a p24δ knockout strain (Tg*Δp24δ*) in RH*Δku80* to investigate its functions, aiming to demonstrate the significance of p24δ in parasite proliferation and virulence.

## 2. Results

### 2.1. Characterization of p24 Protein in T. gondii

**Figure 1:**
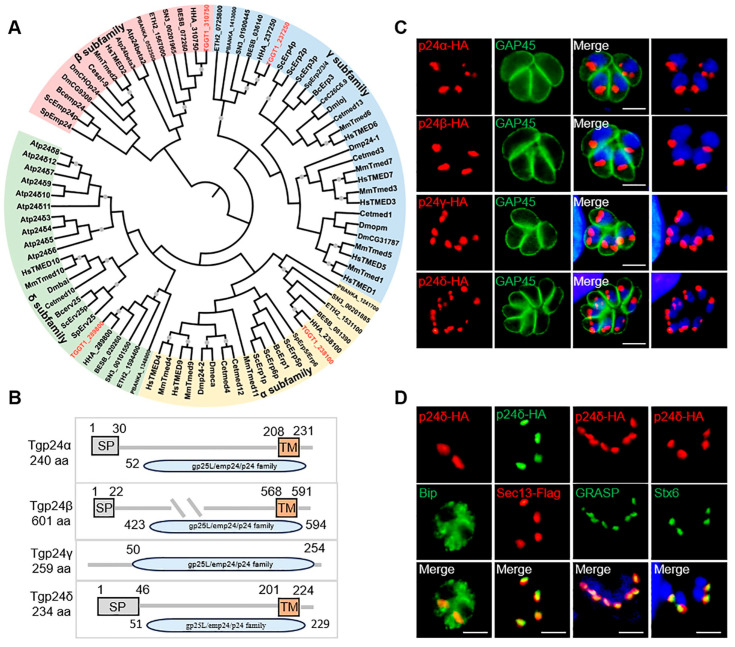
Localization and phylogenetic analysis of p24 family proteins in tachyzoites of *T. gondii*. (**A**) Phylogenetic tree showing p24 proteins across various species, including animals, plants, fungi, and apicomplexans. Species abbreviations: Hs, *Homo sapiens*; Mm, *Mus musculus*; Dm, *Drosophila melanogaster*; Ce, *Caenorhabditis elegans*; At, *Arabidopsis thaliana*; Sc, *Saccharomyces cerevisiae*; Sp, *Schizosaccharomyces pombe*; TG, *Toxoplasma gondii*; ET, *Eimeria tenella*; NC, *Neospora caninum*; HH, *Hammondia*; BESB: *Besnoitia besnoiti*; PB, *Plasmodium berghei*. (**B**) Conserved domains in *T. gondii* p24 proteins are shown in their amino acid sequences. SP: Signal Peptide, TM: Transmembrane Domain. (**C**) Endogenous tagging revealed that Tgp24α, Tgp24β, Tgp24γ, and Tgp24δ localize in punctate or sheet-like structures above the parasite nucleus. Red, mouse anti-HA antibody staining for Tgp24 proteins. Green, TgGAP45 staining for the parasite membrane. Blue, DAPI staining for the tachyzoite nucleus. Scale bars, 2 μm. (**D**) Immunofluorescence showed that Tgp24δ-3HA co-localizes with the endoplasmic reticulum exit site (ERES) protein Sec13 (red) and the cis-Golgi marker GRASP (green). TgSec13-Flag parasites were stained with mouse anti-Flag antibodies (red). Additional markers: mouse anti-Bip (green), mouse anti-GRASP (green), and rabbit anti-Stx6 (green) served as ER, cis-, and trans-Golgi markers, respectively. Scale bars, 2 μm.

We utilized BLAST analysis with the p24 protein sequence from *S. cerevisiae* to search the ToxoDB database (www.toxodb.org, accessed on 3 September 2021) and successfully identified four proteins that contain the p25L/emp24/p24 family domain. Subsequently, we constructed a phylogenetic tree for the p24 family proteins using the neighbor-joining (NJ) method. The tree revealed that the four p24 proteins of *T. gondii* belong to the α, β, γ, and δ subfamilies (Table S1). Moreover, homologous p24 proteins from various apicomplexan protozoa, including *Neospora caninum*, *Hammondia*, *Eimeria tenella*, *Plasmodium*, *Besnoitia besnoiti*, and *Sarcocystis*, clustered together on the same branch of the phylogenetic tree, and these species have a similar number of p24 proteins (Figure 1A). Analysis with PROSITE PROFILES showed that all four proteins contain a conserved p25L/emp24/p24 family domain. Except for Tgp24γ, the other three p24 proteins contain both a signal peptide and a transmembrane domain (Figure 1B and Figure S1).

The deletion of the *ku80* gene in the *T. gondii* RH strain significantly enhances the efficiency of homologous gene recombination during gene editing [22]. To determine the localization of Tgp24s, we inserted a hemagglutinin (HA) epitope tag at the C-terminus of the open reading frame (ORF) of RH*Δku80* Tgp24s (Figure S2). IFA showed that the four p24 proteins formed punctate or sheet-like structures above the parasite nucleus (Figure 1C). To further confirm the specific localization of Tgp24δ, we performed co-localization analysis between Tgp24δ-HA and various markers. These markers included the endoplasmic reticulum (ER) marker Bip, the ER exit site (ERES) protein TgSec13, the cis-Golgi marker GRASP, and the trans-Golgi marker Stx6 [23,24,25,26]. The results showed that Tgp24δ co-localized with TgSec13 and GRASP, suggesting its localization at the ER–Golgi apparatus (Figure 1D).

Digitonin is a detergent that permeabilizes the cytoplasm without affecting organelles and serves as a semi-permeable reagent [27]. To determine whether the C-terminal of Tgp24δ associates with the cytoplasmic exterior or interior, we performed IFA after permeabilizing the sample with various concentrations of digitonin, using BiP as a marker for endoplasmic reticulum permeabilization. BiP is a chaperone protein localized in the ER [23]. At a digitonin concentration of 100 μM, Tgp24δ and GRA1 were detected, but BiP was not. These results suggest that the C-terminus of Tgp24δ likely faces the parasite’s cytoplasm (Figure S3).

**Figure 2:**
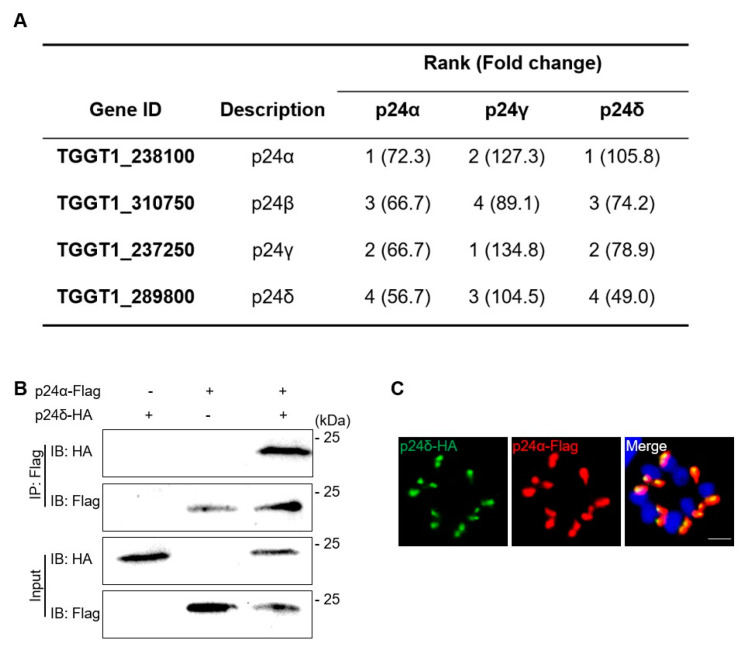
Interaction of Tgp24 family proteins (Tgp24α, Tgp24β, Tgp24γ, and Tgp24δ) in *T. gondii*. (**A**) Immunoprecipitation (IP) assays were conducted to identify proteins interacting with Tgp24-3HA subfamilies (Tgp24α, Tgp24γ, and Tgp24δ), using the parental line RH*Δku80* as a control. The table displays Tgp24α, Tgp24β, Tgp24γ, and Tgp24δ detected by mass spectrometry, with hits ranked by fold changes comparing the mass spectrometry area of Tgp24-3HA subfamilies to the parental line. (**B**) The interaction between Tgp24δ and Tgp24α was confirmed by co-immunoprecipitation from parasites co-expressing endogenous Tgp24δ-3HA and Tgp24α-3Flag. Total lysed proteins (Input) and beads with precipitated complexes (IP panels) were probed with mouse anti-HA and mouse anti-Flag to confirm efficient pulldown of the bait protein. (**C**) IFA showed co-localization of endogenously tagged Tgp24δ-3HA (green) and Tgp24α-3Flag (red). Scale bars, 2 μm.

To investigate potential interactions among Tgp24 protein subfamilies in *T. gondii*, we immunoprecipitated lysates from Tgp24α-3HA, Tgp24γ-3HA, and Tgp24δ-3HA strains with anti-HA-conjugated magnetic beads, which were subjected to mass spectrometry (Table S2). The mass spectrometry results showed that Tgp24α, Tgp24β, Tgp24γ, and Tgp24δ interact with each other (Figure 2A,B). Subsequently, we successfully constructed a double-tagged strain of Tgp24α and Tgp24δ, and IFA analysis showed co-localization between Tgp24α and Tgp24δ (Figure 2C).

### 2.2. Tgp24δ Plays Important Roles in Parasite Growth

**Figure 3:**
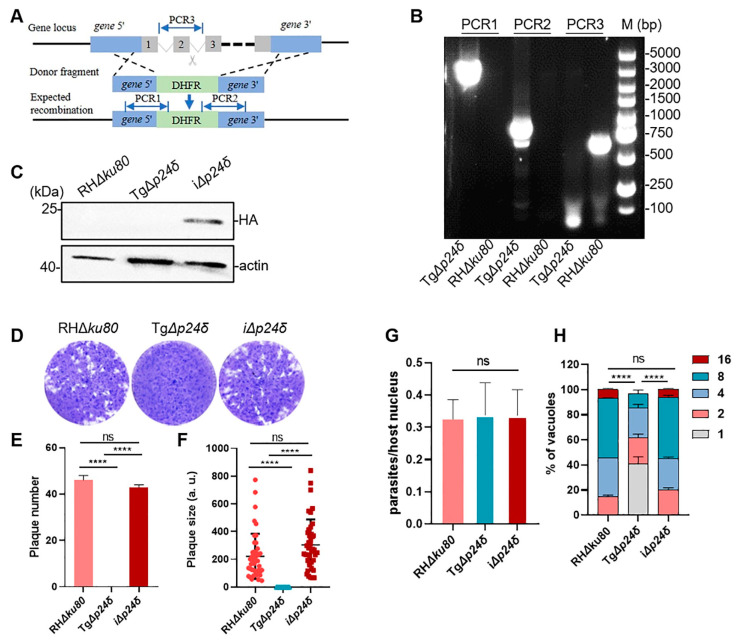
Phenotypic analyses of Tg*Δp24δ* parasite. (**A**) Schematic representation of the CRISPR/Cas9 strategy for deleting the Tg*p24δ* gene, where a DHFR cassette was inserted to replace the Tgp24δ coding sequence. (**B**) Verification of Tg*p24δ* gene deletion by PCR: PCR1 and PCR2 confirm 5’ and 3’ homologous recombination events, respectively; PCR3 verifies successful loss of the Tg*p24δ* gene. (**C**) Western blotting was used to confirm the correct construction of the i*Δp24δ* strain. Bands of 3 × HA-tagged Tgp24δ were detected using mouse anti-HA (**upper panel**), with mouse anti-actin as a loading control (**lower panel**). (**D**) A plaque assay was performed by infecting HFFs with RH*Δku80*, Tg*Δp24δ*, and i*Δp24δ* parasites for 7 days, showing that plaque formation by the Tg*Δp24δ* strain was almost completely inhibited. (**E**,**F**) Quantitative analysis of plaque numbers and sizes produced by each strain revealed a significant reduction in plaque formation by Tg*Δp24δ* strain, which was rescued in i*Δp24δ* strain. (**G**) Invasion assays assessing Tg*Δp24δ* parasite ability to invade host cells, showing comparable invasion efficiency across strains. (**H**) Quantification of Tg*Δp24δ* replication after 24 h growth in HFFs, indicating the ratios of parasites per vacuole (*n* ≥ 100 vacuoles per replicate), demonstrating a significant reduction in replication. Mean ± SD values for three independent experiments, each performed in triplicate. Statistical analysis by unpaired *t*-test or two-way ANOVA, **** *p* < 0.0001; ns, not significant.

To assess the functional roles of Tgp24s in *T. gondii* infection, we used CRISPR/Cas9 to generate complete knockout mutants by replacing the Tg*p24* gene in RH*Δku80* with a DHFR cassette (Figure 3A). A lower phenotype score implies greater importance of the gene for parasite fitness [28]. Due to the low phenotypic values of the four genes (−4.44 for p24α, −6.1 for p24β, −3.51 for p24γ, and −3.23 for p24δ), we were only able to obtain complete knockout mutants for p24δ, indicating that p24δ is crucial for parasite survival. PCR and Western blot results confirmed the successful deletion of Tgp24δ at both the DNA and protein levels (Figure 3B,C). The plaque assay showed a significant reduction in both the number and area of plaques in the Tg*Δp24δ* strain compared to the RH*Δku80* strain (Figure 3D–F). The plaque assay represents the comprehensive abilities of parasites for invasion, replication, and egress. The significant reduction in plaque formation suggests potential defects at one or more stages of the *T. gondii* lytic cycle. Subsequently, we assessed the invasion and replication abilities of Tg*Δp24δ* parasites. We found that the deletion of Tgp24δ did not significantly affect parasite invasion into host cells (Figure 3G). However, the replication ability of Tg*Δp24δ* was significantly decreased compared to the RH*Δku80* strain (Figure 3H). To confirm these findings, we constructed a Tgp24 complementary strain. In this strain, the Tg*UPRT* locus was replaced with the HA-tagged coding sequence, named i*Δp24δ*. Protein expression was confirmed by Western blotting (Figure 3C and Figure S4C). The phenotypic defects of Tg*Δp24δ* parasites were successfully rescued, confirming the phenotypic changes caused by Tgp24δ deletion (Figure 3D–H).

### 2.3. Tgp24δ Knockout Influences the Differenation from Tachyzoite to Bradyzoite in Type I T. gondii

**Figure 4:**
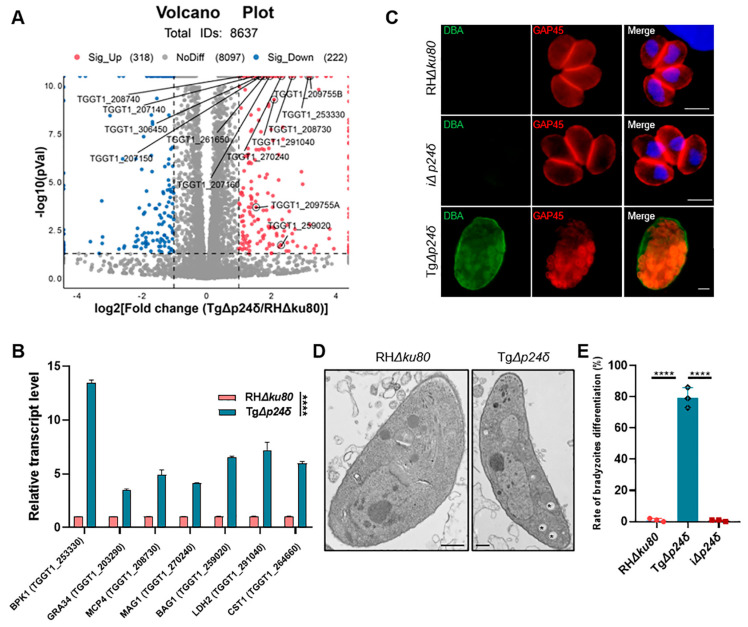
Disruption of Tgp24δ induces the formation of the bradyzoite-specific cyst wall. (**A**) Volcano plots illustrating the significant fold changes in the transcriptome analysis of Tg*Δp24δ* compared to the RH*Δku80* strain. Red and blue dots indicate upregulated (fold changes > 2, *p* < 0.05) and downregulated (fold changes < –2, *p* < 0.05) genes, respectively. The upregulated bradyzoite-specific proteins were highlighted in the volcano plots. (**B**) The transcriptomic findings were validated through quantitative PCR (qPCR) analysis. The relative transcription levels of the targeted genes in the Tg*Δp24δ* and RH*Δku80* strains were normalized to Tg*actin*. Data are presented as means ± SDs from three independent experiments, with statistical differences analyzed using the unpaired *t*-test. **** *p* < 0.0001 indicates a significant difference. (**C**) The cyst wall was stained to assess bradyzoite differentiation status in the parental parasite RH*Δku80*, knockout Tg*Δp24δ* clone, and complemented clone i*Δp24δ* parasites. Infected host cells were cultured under standard conditions for 72 h. The cyst wall was stained with DBA-conjugated Alexa Fluor 488 (green), while parasites were stained with rabbit anti-GAP45 (red). Scale bars, 2 μm. (**D**) Transmission electron micrographs comparing RH*Δku80* and Tg*Δp24δ* parasites. The longitudinal section of the control sample showed a morphologically normal tachyzoite (left). Tg*Δp24δ* parasites contained numerous cytosolic vesicles (indicated by asterisks). Scale bars, 500 nm. (**E**) Quantitative analysis of the cyst wall-positive rate is presented. Each sample included a minimum of 100 counted vacuoles, and the proportion of cyst wall-positive vacuoles is presented. The indicated strains were compared to the RH*Δku80* strain. Data are presented as means ± SDs from three independent experiments, each performed in triplicate; statistical analysis was conducted using the unpaired *t*-test, with **** *p* < 0.0001 indicating significance.

To gain a deeper understanding of the function of Tgp24δ, we employed RNA-seq to examine transcriptional changes in Tg*Δp24δ* tachyzoites compared to RH*Δku80* tachyzoites. Transcriptional analysis identified 501 differentially regulated genes between Tg*Δp24δ* and RH*Δku80*. Specifically, 295 genes were upregulated in Tg*Δp24δ*, and 206 were upregulated in RH*Δku80*, with the criteria of a *p*-value < 0.05 and a |log2FC| ≥ 1. The results showed that under normal culture conditions, the transcription levels of the bradyzoite-specific genes *bag1* (TGGT1_259020), *ldh2* (TGGT1_291040), *bpk1* (TGGT1_253330), *cst1* (TGGT1_264660), *mcp3* (TGGT1_208740), and *mcp4* (TGGT1_208730) were significantly higher in Tg*Δp24δ* than in RH*Δku80* (Figure 4A) [29]. The qPCR results were consistent with the transcriptome analysis, indicating that the deletion of Tgp24δ affects the transcription of these genes (Figure 4B).

The distinct transcriptional differences between Tg*Δp24δ* and RH*Δku80* tachyzoites prompted us to explore the role of Tgp24δ in tachyzoite–bradyzoite regulation and the differentiation rate at 72 h post-infection (Figure 4C). IFA analysis showed cyst wall staining in the Tg*Δp24δ* strain, even under normal culture conditions (Figure 4D). The bradyzoite formation rate in the Tg*Δp24δ* strain reached 79.3%, while there were fewer than 1% CST1-positive vacuoles in the parental RH*Δku80* (Figure 4E). Additionally, transmission electron microscopy was used to compare the morphological changes of Tg*Δp24δ* parasites and RH*Δku80* parasites within the parasitophorous vacuole (PV). Numerous cytosolic vesicles were observed in Tg*Δp24δ* parasites, which resembled the amylopectin granules previously reported in bradyzoites [13]. These results were consistent with the high expression levels of bradyzoite-specific genes observed in the transcriptional analysis. This suggests that Tgp24δ may play an important role in tachyzoite growth.

### 2.4. Comparative Proteomic Analysis Reveals Upregulated Expression of Bradyzoite-Specific Proteins in TgΔp24δ Parasites

**Figure 5:**
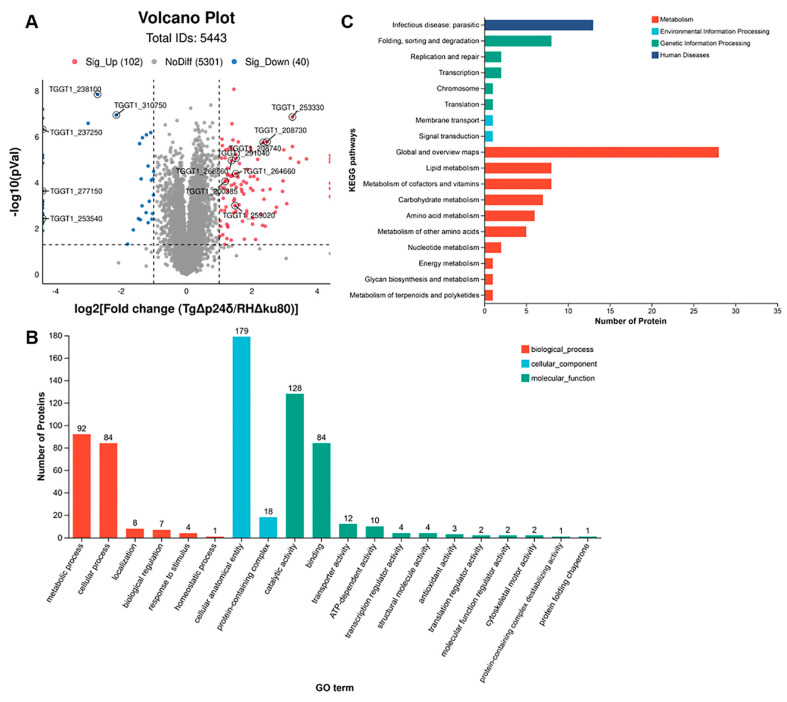
Functional annotation of differentially expressed proteins between RH*Δku80* and Tg*Δp24δ* parasites. (**A**) Volcano plots showed significant fold changes in the proteomic analysis of the Tg*Δp24δ* strain compared with the RH*Δku80* strain. (**B**) Enriched GO terms for differentially regulated proteins as shown across cellular component, biological process, and molecular function categories for Tg*Δp24δ* vs. RH*Δku80*. (**C**) Bar plot illustrating KEGG classification focusing on the Metabolism, Environment Information Processing, Genetic Information Processing, and Human Diseases categories.

Recent studies have identified multiple factors involved in the spontaneous transformation of tachyzoites into bradyzoites. In this study, we aimed to explore the mechanism of tachyzoite-to-bradyzoite transformation in the Tg*Δp24δ* strain through proteomic analysis. Proteomic analysis identified 103 upregulated and 40 downregulated proteins in the Tg*Δp24δ* strain, with the criteria of a *p*-value < 0.05 and a |log2FC| ≥ 1 (Figure 5A). Interestingly, the loss of Tgp24δ led to a significant reduction in the expression levels of Tgp24α, Tgp24β, and Tgp24γ. Consistent with the transcriptional findings, several bradyzoite stage-specific proteins were highly expressed in the Tg*Δp24δ* strain compared to the RH*Δku80* strain. Moreover, proteomic analysis revealed increased expression levels of key transcriptional regulators involved in bradyzoite development, including BFD1 [30].

We conducted GO enrichment analysis to explore the functions of 143 differentially abundant proteins across the cellular component (CC), biological process (BP), and molecular function (MF) categories (Figure 5B). In the cellular component category, the enriched GO terms mainly involved proteins related to intracellular membrane-bound organelles, membranes, and various protein-containing complexes. For the biological process category, the top five enriched terms were “metabolic process”, “cellular process”, “localization”, “biological regulation”, and “response to stimulus”. In the molecular function category, the most abundant GO terms among significantly up- or downregulated proteins were “catalytic activity”, “binding”, “transporter activity”, and “ATP-transcription regulator activity”.

To better understand the metabolic pathways affected by Tgp24δ deletion, we classified the differentially expressed proteins into four categories using the KEGG databases: “Human Diseases”, “Genetic Information Processing”, “Environment Information Processing” and “Metabolism”. These categories were further divided into 18 subcategories, with the “Metabolism” category containing the largest number of identified subcategories. Among the 10 subcategories of “Metabolism”, “Global and overview maps” had the highest enrichment of differentially expressed proteins, followed by “Lipid metabolism”, “Metabolism of cofactors and vitamins”, and “Carbohydrate metabolism” (Figure 5C). Tachyzoites and bradyzoites exhibit distinct metabolic characteristics to meet their carbon demands [11]. The alterations in metabolic pathways caused by the deletion of Tgp24δ may be associated with the tachyzoite-to-bradyzoite transformation.

### 2.5. Tgp24δ Is an Essential Virulence Factor

**Figure 6:**
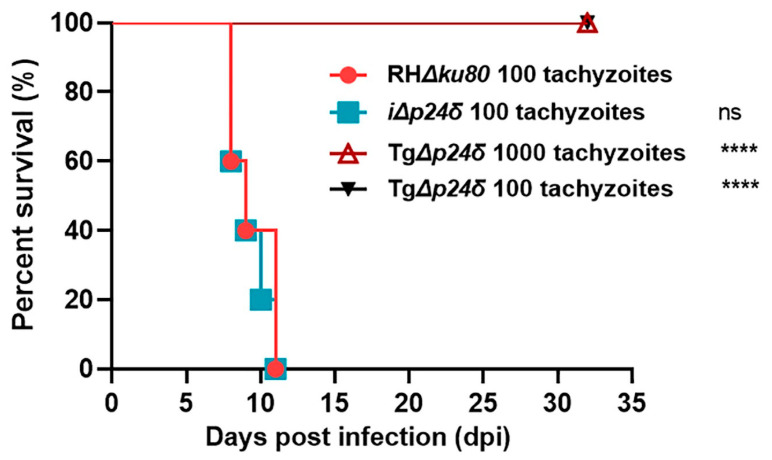
The Tgp24δ is an essential virulence factor. Each group of mice (n = 5 per strain) was intraperitoneally (i.p.) injected with 100 tachyzoites of RH*Δku80*, Tg*Δp24δ*, or i*Δp24δ* or 1000 tachyzoites of Tg*Δp24δ* and then monitored for 30 days. The indicated strains were compared to the RH*Δku80* strain. All Tg*Δp24δ*-infected mice remained alive and exhibited no clinical signs of illness. Data are presented as means ± SDs, and statistical differences were analyzed by the log-rank test (Mantel–Cox test). **** *p* < 0.0001 indicates highly significant differences. ns indicates not significant.

To assess the effect of Tgp24δ deletion on the virulence of the type Ι RH strain, groups of BALB/c mice (*n* = 5 for each group) were intraperitoneally (i.p.) injected with 100 or 1000 tachyzoites suspended in 200 μL of phosphate-buffered saline (PBS) for each strain. In accordance with the results of parasite proliferation in vitro, all mice infected with RH*Δku80* or i*Δp24δ* parasites died within 8 to 12 days (Figure 6). In contrast, mice that were infected with 100 or 1000 Tg*Δp24δ* tachyzoites showed no clinical symptoms, and all of them survived. Even though the Tg*Δp24δ* strain had a high cyst formation rate in vitro, the 529 bp gene was undetectable in brain tissue from surviving mice at 35 days post-infection. In conclusion, RH*Δku80* parasites with the deletion of Tgp24δ demonstrated decreased growth and virulence in mice and failed to differentiate from tachyzoites to bradyzoites in the host brain.

### 2.6. Influence of Tgp24δ Deletion on Protein Transport in Parasites

**Figure 7:**
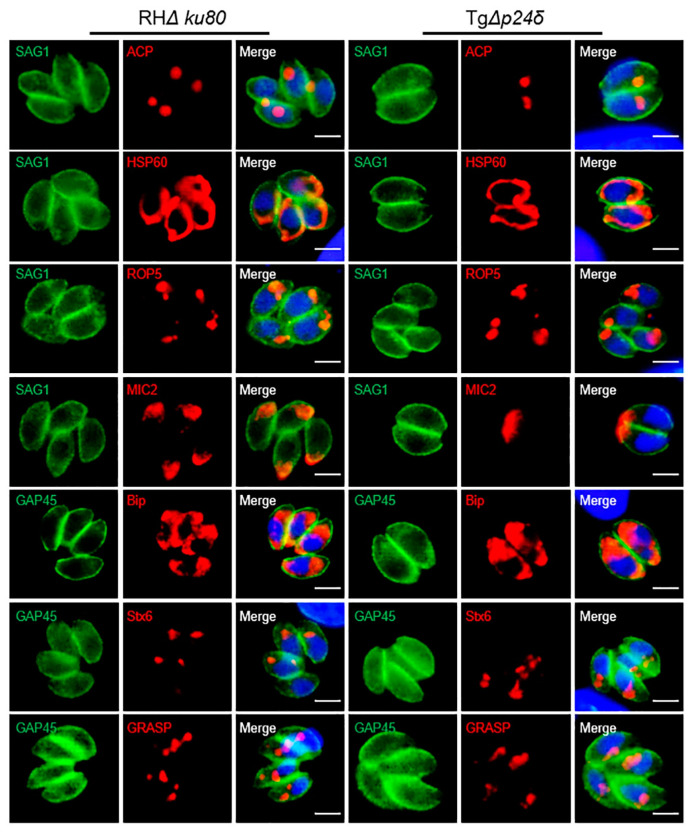
The absence of Tgp24δ does not affect the transport of specific proteins in *T. gondii*. IFA was performed on RH*Δku80* and Tg*Δp24δ* parasites. SAG1 (green), GAP45 (green), and DAPI (blue) stains were used to label the parasites and nuclei (DNA). Anti-ACP, anti-HSP60, anti-ROP5, anti-MIC2, anti-Bip, anti-Stx6, and anti-GRASP were used to stain the apicoplasts, mitochondria, rhoptries, micronemes, endoplasmic reticulum (ER), trans-Golgi, and cis-Golgi, respectively. Scale bars, 2 μm.

Nearly all secreted proteins of *T. gondii* reach their destinations through the ER and Golgi pathways [31]. To explore the role of Tgp24δ in protein trafficking, we examined several classical secretory proteins, including MIC2, GRA1, ROP5, and SAG1, a GPI-anchored protein. Deletion of Tgp24δ did not affect the localization of these proteins (Figure 7). Additionally, we observed that deficiency of Tgp24δ did not alter the localization of ER, Golgi, or apicoplast proteins (Figure 7). These findings suggest that Tgp24δ is not involved in mediating the transport of these proteins.

## 3. Discussion

The p24 family proteins are well known for their function as cargo receptors in both anterograde and retrograde vesicular transport processes between the ER and Golgi [14]. Phylogenetic analysis has revealed that the p24 family proteins are widely distributed across diverse organisms, including animals, plants, fungi, and apicomplexans. The p24 protein family can be classified into four subfamilies: α, β, γ, and δ. In apicomplexans, four distinct p24 proteins exist, each belonging to a separate subfamily. In *S. cerevisiae*, p24 family members assemble into a heteromeric complex composed of Emp24p, Erv25p, Erp1p, and Erp2p [32,33]. The loss of Emp24p and Erv25p destabilizes the p24 protein complex, causing selective trafficking defects in secretory proteins as they move from the ER to the Golgi [18,19]. In this study, mass spectrometry data indicated interactions among the four isoforms of p24 proteins in *T. gondii.* The observed co-localization between Tgp24α and Tgp24δ was likely due to our inability to successfully construct double-tagged strains for all pairs of isoforms. Despite multiple attempts, we were only successful in generating the double-tagged strain for Tgp24α and Tgp24δ. IFA results showed that Tgp24δ and Tgp24α co-localized within the ER–Golgi system in *T. gondii*, mirroring the localization patterns of Emp24p in *S. cerevisiae*. Proteomic analysis showed that loss of p24δ significantly reduced the expression levels of the other three p24 proteins. This finding suggests that the p24 family protein of *T. gondii* may participate in the early secretory pathway through complex formation.

Furthermore, we found that the number of p24 family proteins in apicomplexan parasites is significantly lower compared to that in other species. In *Homo sapiens*, *Drosophila melanogaster*, and *Caenorhabditis elegans*, there are 8–10 p24 family proteins belonging to the four subfamilies [21,34,35]. In *Arabidopsis*, there are 12 p24 proteins, but it lacks p24α and p24γ members [36]. In contrast, apicomplexan parasites possess only four p24 proteins (Table S1). The variation in the quantity of p24 proteins indicates that the functions of p24 proteins may vary among species.

The specific functions of the p24 proteins in apicomplexans remain unclear. There are four p24 family proteins in *Plasmodium falciparum*, of which two (PF3D7_0526900 and PF3D7_0422100) are essential for liver-stage viability or sporozoite infectivity; however, the exact molecular mechanisms of these p24 proteins during these stages require further study [37]. In this study, we found that the absence of Tgp24δ significantly reduced the replication capacity of *T. gondii* without impairing its invasion ability. The survival curves of the infected mice demonstrated that the Tg*Δp24δ* strain did not cause death in the mice, suggesting that Tgp24δ is important for parasite virulence. However, we were unable to generate knockout mutants of Tgp24α, Tgp24β, and Tgp24γ. This failure likely indicates a lethal phenotype associated with the deletion of these isoforms. This raises the possibility that Tgp24α, Tgp24β, and Tgp24γ may play an even more essential role in parasite survival. Further research should focus on developing alternative experimental strategies, such as conditional knockout systems, to investigate the functions of these isoforms. Moreover, homologous p24 proteins from diverse apicomplexan protozoa, such as *N. caninum*, *P. falciparum*, and *E. tenella*, are clustered together on the same branch of the phylogenetic tree, indicating evolutionary relatedness and potentially conserved functions among these p24 proteins across different apicomplexan parasites.

The key to the virulence of *T. gondii* infection is the secretion of numerous effector proteins. These proteins mediate crucial processes, such as host cell invasion, replication, nutrient acquisition, and immune evasion, in host cells [38]. Secreted proteins of *T. gondii*, including microneme and rhoptry proteins, are transported from the ER through the Golgi apparatus to the apical surface or directly into the host cell via the classic ER–Golgi pathway [31]. However, the deletion of Tgp24δ did not disrupt the localization of various secreted proteins, including MIC2, GRA1, ROP5, GPI- anchored protein SAG1, the Golgi apparatus protein Stx6, and the ER protein Bip, in the parasite. This suggests that Tgp24s may be involved in the transport of specific proteins or perform a unique function in *T. gondii* compared to other species.

Tgp24δ and TMED10 are located on the same phylogenetic branch and belong to the δ subfamily, indicating potential functional similarities. Zhang et al. found that TMED10 is required for the secretion of mature IL-1β in both inflammatory and non-inflammatory cells, as demonstrated by protein cross-linking and mass spectrometry [39]. Cross-linking reagents are used to stabilize transient protein interaction, while co-IP mainly detects stable protein interactions [40]. This difference may explain why we were unable to detect the cargo protein of Tgp24δ through IP experiments. Unfortunately, the failure of our cross-linking experiment prevented us from acquiring evidence to confirm the role of Tgp24δ as a cargo receptor in *T. gondii*.

The slower proliferation rate of *T. gondii* is a characteristic feature of tachyzoite-to-bradyzoite conversion. In this study, we used RNA-seq and proteomic analyses to determine that a variety of bradyzoite stage-specific genes, such as *bag1*, *ldh2*, *bpk1*, and *srs9*, were upregulated in the Tg*Δp24δ* strain compared to the RH*Δku80* strain. Furthermore, the rate of bradyzoite formation in the Tg*Δp24δ* strain reached 79.3%. These results suggest that the Tg*Δp24δ* strain maintains a high bradyzoite formation rate even under conditions typically favoring tachyzoite replication. BFD1, a myb-like transcription factor, is crucial for promoting the transformation of *T. gondii* from tachyzoites to bradyzoites [30]. ApiAP2 transcription factors have both activating and repressive roles in gene expression during the tachyzoite–bradyzoite transition [41]. Unlike these transcriptional regulators, Tgp24δ appears to have a more direct impact on the replication and virulence of the parasite. However, it is possible that Tgp24δ participates in parallel pathways to orchestrate the complex processes associated with the tachyzoite–bradyzoite transition. Further research is needed to clarify the role of Tgp24δ in differentiation and its functional relationships with other regulatory factors.

## 4. Materials and Methods

### 4.1. Proteins and Phylogenetic Analysis

Using the p24 family proteins from *Saccharomyces cerevisiae* as query sequences, we identified the putative proteins for p24 family proteins in *T. gondii* [16]. Phylogenetic analysis and tree construction were performed using MEGA-X 10.0 software. Sequence data from other referenced species were obtained from the GenBank database at the National Center for Biotechnology Information (http://www.ncbi.nlm.nih.gov/, accessed on 3 September 2021).

Signal peptides and transmembrane regions in p24 proteins were predicted using the SignalP-5.0 (https://services.healthtech.dtu.dk/service.php?SignalP-5.0/, accessed on 4 September 2021) and TMHMM v. 2.0 servers (https://services.healthtech.dtu.dk/service.php?TMHMM-2.0/, accessed on 4 September 2021). Conserved functional domains of p24 proteins were predicted using ScanProsite (https://prosite.expasy.org/scanprosite/, accessed on 4 September 2021) and InterPro (https://www.ebi.ac.uk/interpro/, accessed on 4 September 2021).

### 4.2. Parasite and Cell Cultures

RH*Δku80* served as the parental parasite for constructing the transgenic strains. Parasites were cultured and passaged in African green monkey kidney (Vero) cells and human foreskin fibroblasts (HFFs, maintained in our laboratory), using Dulbecco’s modified Eagle’s medium (DMEM) supplemented with 2% fetal bovine serum (FBS) at 37 °C in an incubator with 5% CO_2_.

### 4.3. Generation of Transgenic Strains

All transgenic strains were generated using the clustered regularly interspaced short palindromic repeat (CRISPR)-Cas9 system, as previously described [42,43]. Primer sequences are listed in Table S3. To construct the epitope-tagged strain, we used an online tool (www.toxodb.org, accessed on 3 September 2021) to design sgRNA from the GOI stop codon downstream, covering a sequence of approximately 200–300 bp. The gRNA was integrated as a homologous recombination sequence downstream of U6 and upstream of AMP primers. The pCas9-sgRNA plasmids were constructed using pSAG1:CAS9-U6:sgUPRT as a template. Three fragments were amplified using Cas9-F/R, U6-F/U6-sgRNAxx, and AMP sgRNAxx/AMP-R primers. The three fragments were ligated according to the protocol of the Basic Seamless Cloning and Assembly kit (CU201-02, TransGen Biotech, Beijing, China). C-terminal homologous fragments, containing a 42 bp fragment of the GOI gene and a tagged product, were amplified using pLIC-3HA-DHFR, pL-miniAID-3HA-HXGPRT, or pLIC-3flag-CAT as the template, as previously described [42,43]. Finally, the corresponding fragments (~15 μg) and the specific CRISPR/Cas9 plasmid (~40 μg) were co-transfected into RH*Δku80*. Positive parasites were selected using 25 μg/mL mycophenolic acid (M5255, Sigma-Aldrich, Burlington, MA, USA) and 25 μg/mL 6-xanthine (X4002, Sigma-Aldrich), 3 μM pyrimethamine (46706, Sigma-Aldrich), or 20 μM chloramphenicol (220551, Sigma-Aldrich). Recombinant monoclonal clones were identified using IFA and Western blot analysis.

To construct the Tgp24δ knockout strain (Tg*Δp24δ*), the sgRNA targeting the Tg*p24δ* gene was selected. For the construction of p5’ p24δ-DHFR-3’ p24δ, we amplified the 5’, 3’ homologous fragments of the Tg*p24δ* gene and the DHFR gene from RH*Δku80* genomic DNA and the pLIC-3HA-DHFR plasmid, respectively. The p5’ p24δ-DHFR-3’ p24δ plasmid was constructed and then linearized. The transfection and drug selection were performed according to the method described above. The monoclonal clones were verified by PCR.

To complement the Tgp24δ knockout strain (i*Δp24δ*), the complete Tgp24δ coding region was amplified from the cDNA of the RH*Δku80* parasite and inserted into the p5’ UPRT-3HA-DHFR-3’ UPRT. Homologous fragments and pCas9-gRNA (UPRT) were then co-transfected into the Tg*Δp24δ* strain. After electroporation and selection with floxuridine (FUDR), monoclonal clones were identified by Western blot analysis and IFA.

### 4.4. Indirect Immunofluorescence Assay (IFA) Staining and Western Blotting

For immunofluorescence, the primary antibodies used in this study included mouse anti-HA (1:1000), rabbit anti-HA (Sigma, 1:1000), mouse anti-Flag (Sigma, 1:1000), rabbit anti-Flag (Sigma, 1:1000), mouse anti-SAG1 (1:300), mouse anti-IMC1 (1:300), rabbit anti-IMC1 (1:300), rabbit anti-GAP45 (1:300), rabbit anti-HSP60 (1:500), rabbit anti-ACP (1:500), rabbit anti-ROP5 (1:500), Rabbit anti-MIC2 (1:1000), mouse anti-Bip (1:500), rabbit anti-Stx6 (1:500), and mouse anti-GRASP (1:300) (all maintained in our laboratory) [42]. Parasites were grown in HFF monolayers on coverslips, washed three times with phosphate-buffered saline (PBS; Solarbio, Beijing, China), then fixed with 4% paraformaldehyde for 30 min, permeabilized with 0.25% Triton X-100 (Solarbio, Beijing, China) in PBS for 20 min, and blocked with 3% BSA (M&C Gene, Beijing, China) in PBS for 30 min. Parasites were then incubated with various primary antibodies diluted in 3% BSA at 37 °C for 1 h, washed three times, and subsequently incubated with secondary antibodies (FITC-conjugated goat anti-mouse IgG (H + L), dilution: 1:100, or Cy3-conjugated goat anti-rabbit IgG (H + L), dilution: 1:100; Proteintech, Wuhan, China) at 37 °C for another hour. For cyst wall staining, FITC-conjugated Dolichos biflorus lectin (Vector laboratories Inc., Newark, CA, USA) was used. Finally, an antifade mounting medium containing DAPI (Solarbio, Beijing, China) was applied to seal the coverslips and glass slides. Imaging of the parasites was conducted using a fluorescence microscope (Olympus, Tokyo, Japan).

For Western blotting, parasites were harvested and lysed in RIPA buffer containing a protease inhibitor cocktail on ice. Proteins were separated by SDS-PAGE and transferred to a PVDF membrane (Millipore, Burlington, MA, USA). Immunoblotting was performed using primary antibodies, including mouse anti-HA (Sigma, 1:5000), mouse anti-flag (Sigma, 1:5000), and mouse anti-actin (1:5000, maintained in our lab). HRP-conjugated antibodies (1:5000) were applied as secondary antibodies to detect these primary antibodies. Images were visualized using a Tano imaging system (Shanghai Tianneng, Shanghai, China).

### 4.5. Plaque Assay

Freshly released tachyzoites were inoculated at 150 tachyzoites per well on 12-well plates with HFF monolayers at 90% confluence. After incubation at 37 °C with 5% CO_2_ for 6–7 days, the HFF monolayers were fixed with 4% paraformaldehyde for 15 min, stained with 1% crystal violet for 30 min at room temperature, and then gently washed with PBS. Plaque number and size were analyzed from images obtained with a scanner as previously described [42,43]. All plaque assays were performed in triplicate.

### 4.6. Invasion Assay

The invasion ability of tachyzoites was assessed as previously described [42,43]. Briefly, tachyzoites of RH*Δku80*, Tg*Δp24δ*, and i*Δp24δ* were harvested and counted. A total of 1 × 10^6^ parasites were added to coverslips placed in the bottom of 12-well plates with monolayers of HFF cells and allowed to invade for 40 min at 37 °C. The culture medium was removed, and the monolayers were gently washed three times with PBS to remove unattached tachyzoites. The cells were fixed with 4% paraformaldehyde. Non-invaded parasites were stained with mouse anti-SAG1, followed by three PBS washes to remove unbound antibodies. Before the second antibody incubation, cells were permeabilized with 0.25% Triton X-100. Invaded parasites were then detected with rabbit anti-IMC1 (maintained in our lab). FITC goat anti-mouse, Cy3 goat anti-rabbit, and Hoechst (Sigma, 1:100) were used to stain the non-invaded parasites, invaded parasites, and the host cell nuclei, respectively. Independent invasion assays were performed in triplicate. Twenty-fields per coverslip were quantified, and the number of invaded parasites was calculated by subtracting the number of attached parasites from the total parasite count across three independent experiments.

### 4.7. Intracellular Replication Assay

A total of 5 × 10^5^ freshly released tachyzoites of each strain were inoculated onto HFFs grown on 12-well plate coverslips for 1 h. The tachyzoites that remained attached to the cell surface were washed three times with PBS and supplemented with fresh medium and incubated for 24 h at 37 °C with 5% CO_2_. The cells were then washed with PBS and fixed with 4% formaldehyde. IFA was performed to stain the parasite membrane with rabbit anti-TgGAP45 (1:300) as the primary antibody and FITC-conjugated goat anti-rabbit IgG (H + L) as the secondary antibody. The number of parasites per vacuole was counted, and 100 parasitophorous vacuoles (PVs) were counted in several random fields using a fluorescence microscope. The experiment was performed three times.

### 4.8. Co-Immunoprecipitation and Mass Spectrometry

Tachyzoites (1 × 10^9^ parasites) from Tgp24α-HA, Tgp24γ-HA, Tgp24δ-HA, and RH*Δku80* strains were lysed with cell lysis buffer for Western blotting and immunoprecipitation (IP) (P0013; Beyotime, Shanghai, China). After 30 min on ice, the lysates were centrifuged at 14,000 g for 20 min at 4 °C, and the supernatants were incubated with anti-HA agarose beads (P2185S; Beyotime, Shanghai, China) overnight at 4 °C under rotation. After five washings with TBS, immunoprecipitates were eluted with 100 μL of elution buffer containing protein loading buffer (P0015; Beyotime, Shanghai, China) at 95 °C for 10 min. The samples were analyzed by SDS-PAGE, stained with Coomassie blue R250 in 45% methanol and 10% glacial acetic acid, and then destained in 25% methanol and 8% glacial acetic acid. Protein lanes were excised and sent to Shanghai Applied Protein Technology Co., Ltd. (Shanghai, China). for mass spectrometry analysis. RH*Δku80* control lines and HA fusion lines were conducted in parallel, each with two technical and biological replicates. Raw data were searched against the *T. gondii* database (https://toxodb.org, accessed on 10 May 2023). Peptides and proteins were identified with a 1% false-discovery rate (FDR), and proteins with single modification sites, contaminants, or reversed sequences were removed. We retrieved data, including *p*-values and fold changes (Table S2), for assessing the protein–protein interactions.

### 4.9. RNA-Seq Analysis and DIA Quantitative Proteomic Analysis

Tachyzoites of the RH*Δku80* and Tg*Δp24δ* strains were used to infect T-75 cell culture flasks containing HFF monolayers. To account for the lower replication rate of the mutant strain, Tg*Δp24δ* was cultured for a longer period than the wild type. Cells infected with WT or Tg*Δp24δ* tachyzoites were cultured at 37 °C for 36 or 48 h, respectively. After washing with cold PBS, parasites were harvested by passing through 22G needles and filtration through 3.0 μm polycarbonate membranes. For transcriptional analysis, the parasites were immediately resuspended in cold Trizol (Solarbio, Beijing, China) for total RNA extraction according to the manufacturer’s protocol. Then, RNA quality was determined by a 5300 Bioanalyser (Agilent) and quantified using an ND-2000 (NanoDrop Technologies, Wilmington, DE, USA). Only high-quality RNA samples were used for library construction and sequencing by Shanghai Majorbio Bio-pharm Biotechnology Co., Ltd. (Shanghai, China). The raw paired-end reads were trimmed and quality-controlled using fastp (https://github.com/OpenGene/fastp, accessed on 14 May 2024) with default parameters. Clean reads were aligned to the reference genome (https://toxodb.org/toxo/app, accessed on 3 May 2024) using HISAT2 software (http://ccb.jhu.edu/software/hisat2/index.shtml, accessed on 16 May 2024). The mapped reads of each sample were assembled using StringTie in a reference-based approach, and the expression level of each transcript was calculated according to the transcripts per million reads (TPM) method. Differential expression analysis was performed using DESeq2 (http://bioconductor.org/packages/stats/bioc/DESeq2/, accessed on 22 May 2024). Different expression genes (DEGs) with a *p*-value < 0.05 and a |log2FC ≥ 1| were considered to be significantly differently expressed genes (Table S4).

For proteomic analysis, parasites were harvested as described above and stored at −80 °C. The methods for protein extraction, digestion, DIA mass detection, and protein identification were carried out as previously described [44]. Differentially expressed proteins were identified using thresholds of a *p*-value < 0.05 and a |log2FC ≥ 1| (Table S5).

### 4.10. Quantitative Real-Time PCR

Total RNA of tachyzoites was extracted using TRIZOL^®^ reagent (Invitrogen, Carlsbad, CA, USA) according to the manufacturer’s instructions. RNA quality and concentration were measured using a NanoDrop 2000c spectrophotometer (ThermoFisher, Waltham, MA, USA). Equal amounts of total RNA were reverse transcribed into cDNA using the TransScript One-Step gDNA Remover and cDNA Synthesis SuperMix kit (AT311; TransGen Biotech, Beijing, China) according to the manufacturer’s instructions. Quantitative real-time PCR (qRT-PCR) was then performed on a StepOnePlus™ qRT-PCR system (ThermoFisher) using PerfectStart Green qPCR SuperMix (AQ132; TransGen Biotech, Beijing, China). The total reaction volume was 20 μL, comprising 10 μL of 2 ×PerfectStart Green qPCR SuperMix, 0.4 μL of each 10 μM gene-specific primer, 2 μL of cDNA, and 7.2 μL of ddH_2_O. Three biological replicates were performed for each qRT-PCR experiment. The thermal cycling conditions began with 10 min denaturation at 95 °C, followed by 40 cycles of 15 s at 95 °C and 1 min at 60 °C. Primer sequences are listed in Table S6.

### 4.11. Transmission Electron Microscopy (TEM)

Transmission electron microscopy was performed as previously described [45]. Tachyzoites of RH*Δku80* and Tg*Δp24δ* were cultured for 24 or 36 h, and cells containing PVs were gently collected using a cell scraper. After washing the samples twice with PBS, they were fixed in a solution containing 1% paraformaldehyde and 2.5% glutaraldehyde for at least 2 h, followed by fixation in the dark with 1% osmium acid solution for 2–4 h. The samples were then immersed in a mixture of acetone and Spurr’s resin for 12 h, followed by immersion in pure Spurr’s resin for at least 8 h, and finally polymerized at 70 °C for 14 h. Ultrathin sections, 60 to 80 nm thick, were prepared using a Leica EM UC7 ultramicrotome (Würzburg, Germany) and placed on single-slot grids coated with a Formvar membrane. The ultrathin sections were stained with 2% uranyl acetate and lead citrate. Finally, the sections were examined using a transmission electron microscope (RuliTEMHT7800; Hitachi, Tokyo, Japan) to observe ultrastructural changes.

### 4.12. Assessment of Virulence

To assess the virulence of the transgenic strain relative to the RH*Δku80* strain, six-week-old female BALB/c mice (Vitalriver, Beijing, China) were intraperitoneally (i.p.) injected with 100 or 1000 *T. gondii* tachyzoites in a volume of 100 μL sterile PBS. The mice were housed under specific pathogen-free (SPF) conditions in filter-top cages, with sterile water and food provided. Clinical symptoms were observed every 24 h for up to 30 days, and survival rates were recorded. The virulence of the different strains was compared using survival curves. Three independent experiments were performed.

### 4.13. Statistical Analyses

GraphPad 8.0 was used to analyze the data and generate graphs. All statistical analyses were performed using the Student’s *t*-test or a two-way analysis of variance (ANOVA). Unless otherwise specified, data were based on three independent biology replicates. *p*-values < 0.05 were considered significant.

## Data Availability

The original contributions presented in this study are included in the article/Supplementary Materials. Further inquiries can be directed to the corresponding author.

## Supplementary Materials

The following supporting information can be downloaded at https://www.mdpi.com/article/10.3390/ijms26073331/s1.
