# Supplementary material for: Role of Toxoplasma gondii p24δ in Regulating the Transition from Tachyzoite to Bradyzoite Development
Source: Int J Mol Sci. 2025 Apr 3;26(7):3331. doi: 10.3390/ijms26073331 (PMC11989233; doi:10.3390/ijms26073331)
Supplement: Supplementary file 1 [file ijms-26-03331-s001.zip › sup-table1.pdf]

Table S1 Classification and quantity of p24 family proteins in different species

Species	α	β	γ	δ	Total
Toxoplasma gondii	TGGT1_238100	TGGT1_237250	TGGT1_310750	TGGT1_289800	4
Neospora caninum	NCLIV_015480	NCLIV_051110	NCLIV_054800	NCLIV_042050	4
Plasmodium berghei	PBANKA_1241700	PBANKA_1413000	PBANKA_0522500	PBANKA_1348000	4
Eimeria tenella	ETH2_1531100_mRNA1	ETH2_0725800	ETH2_1567000	ETH2_1594400	4
Hammondia hammondi	HHA_238100	HHA_237250	HHA_310750	HHA_289800	4
Sarcocystis neurona	SN3_00201885	SN3_00201965	SN3_00201965	SN3_00101550	4
Besnoitia besnoiti	BESB_081390	BESB_072260	BESB_036140	BESB_020260	
Saccharomyces cerevisiae	Erp1p, Erp5p, Erp6p	Emp24p	Erp2p, Erp3p, Erp4	Erv25p	8
Schizosaccharomyces pombe	SpErp5/Erp6	SpEmp24	SpErp2/3/4	SpErv25	4
Arabidopsis	0	p24β2, p24β3	0	p24δ3-11	12
Homo Sapiens	TMED4, TMED9	TMED2	TMED1, TMED3, TMED5, TMED6, TMED7	TMED10	9
Drosophila melanogaster	Dmp24-2, Dmeca	DmCHOp24, DmCG9308	Dmloj, Dmp24-1, DmCG31787, Dmopm	Dmbai	9
Caenorhabditis elegans	TMED4, TMED12	SEL-9	TMED13, TMED3, TMED1, C26C6.9	TMED10	8
Mus musculus	MmTmed4, MmTmed9, MmTmed11	MmTmed2	MmTmed1, MmTmed3, MmTmed5, MmTmed6, MmTmed7	MmTmed10	10
