# Supplementary material for: Role of Toxoplasma gondii p24δ in Regulating the Transition from Tachyzoite to Bradyzoite Development
Source: Int J Mol Sci. 2025 Apr 3;26(7):3331. doi: 10.3390/ijms26073331 (PMC11989233; doi:10.3390/ijms26073331)
Supplement: Supplementary file 1 [file ijms-26-03331-s001.zip › Supplementary Section .pdf]

SUPPLEMENTARY MATERIAL

Figure S1

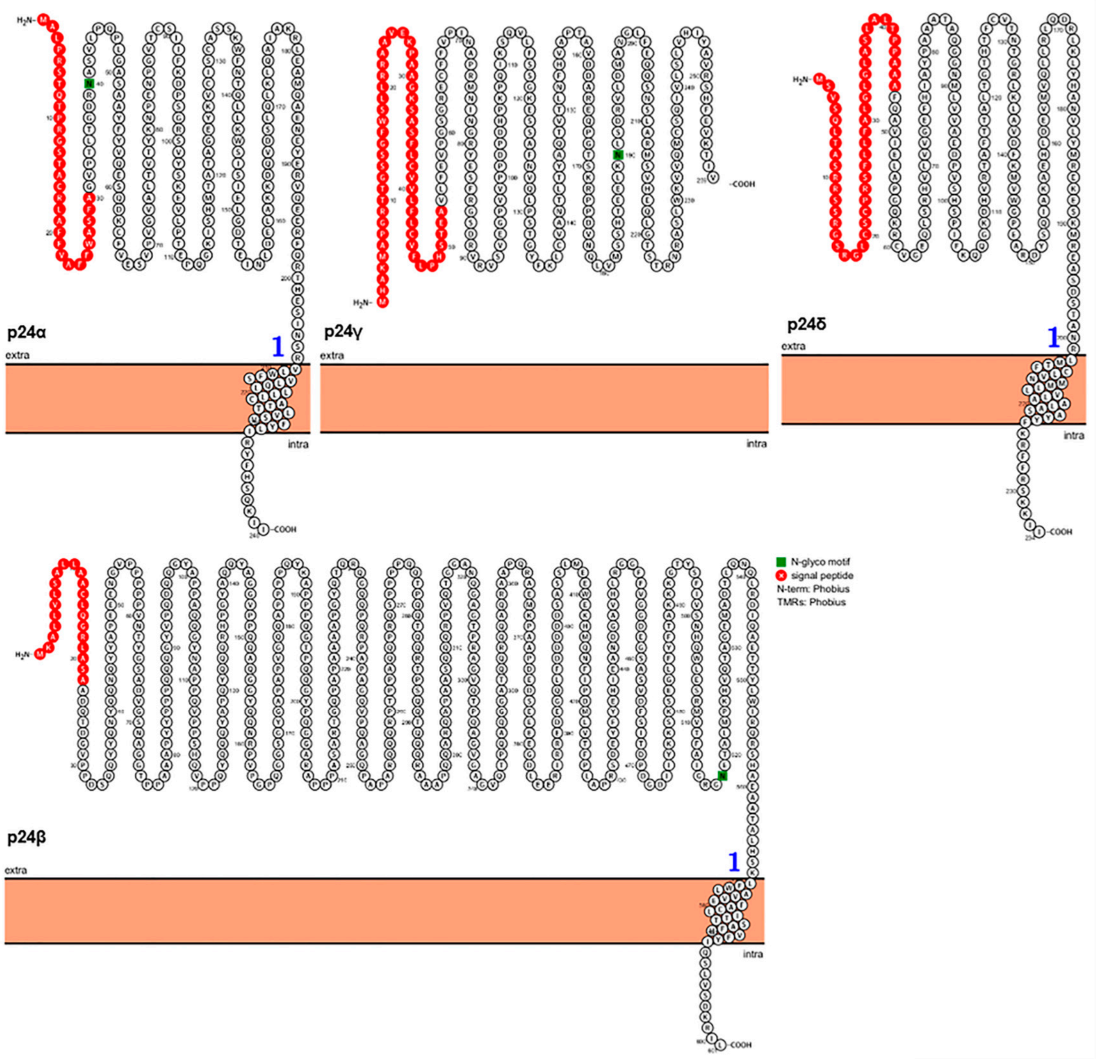

Figure S1. Protter analysis (https://wlab.ethz.ch/protter/#) of Tgp24 family protein amino acid sequences reveal signal peptides in all four proteins. Three proteins (Tgp24α, Tgp24β, and Tgp24δ), except Tgp24γ, present a transmembrane domain.

Figure S2

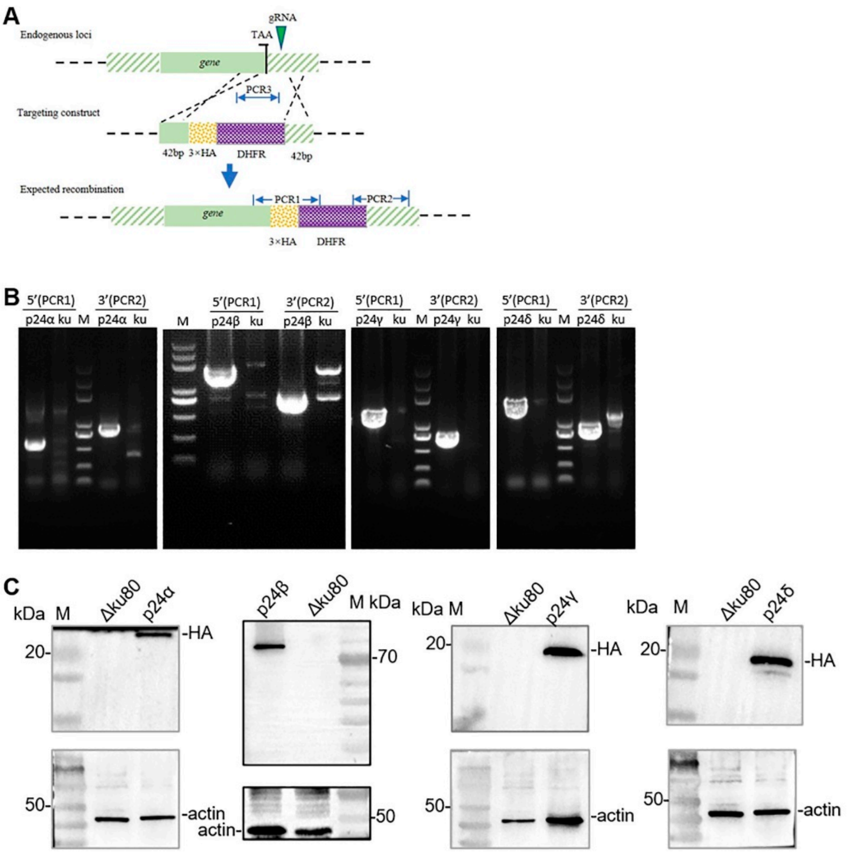

Figure S2. Endogenous tagging of Tgp24 family protein in *T. gondii.* (A) Schematic diagram depicting C-terminal endogenous tagging of the gene of Tgp24 family protein. (B) PCR1 and PCR2 confirmed correct tag integration in the indicated strains. (C) Western blot detected the expression of hemagglutinin (HA)-tagged Tgp24 family protein in the corresponding strains, with Actin as a loading control. The bands of Tgp24α-3HA, Tgp24β-mAID-3HA, Tgp24γ-3HA, and Tgp24δ-3HA were detected at about 27, 77, 29, and 26kDa, respectively.

Figure S3

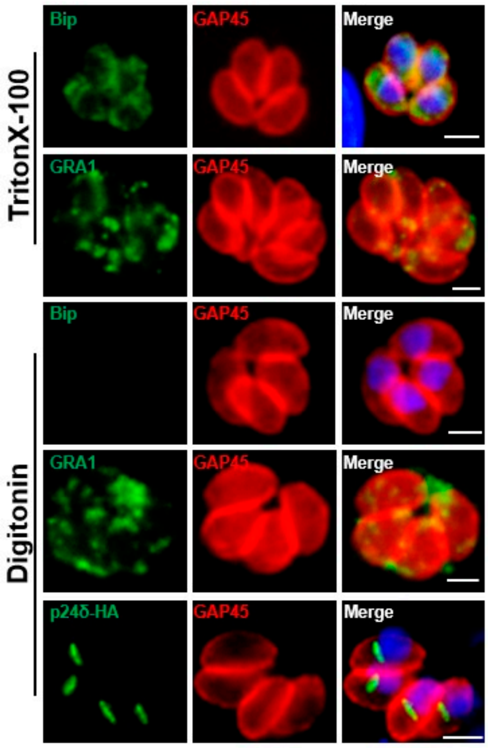

Figure S3. Intracellular parasites of Tgp24δ-3HA and RH*Δku80* strains were fixed and permeabilized with 100 μM digitonin. Complete permeabilization was achieved using 0.25% Triton X-100 as a control. BiP and GRA1 were used as controls for luminal and cytosolic proteins, respectively. IFA showed the C-terminal of Tgp24δ is oriented towards the cytoplasm. Scale bars, 2 μm.

Figure S4

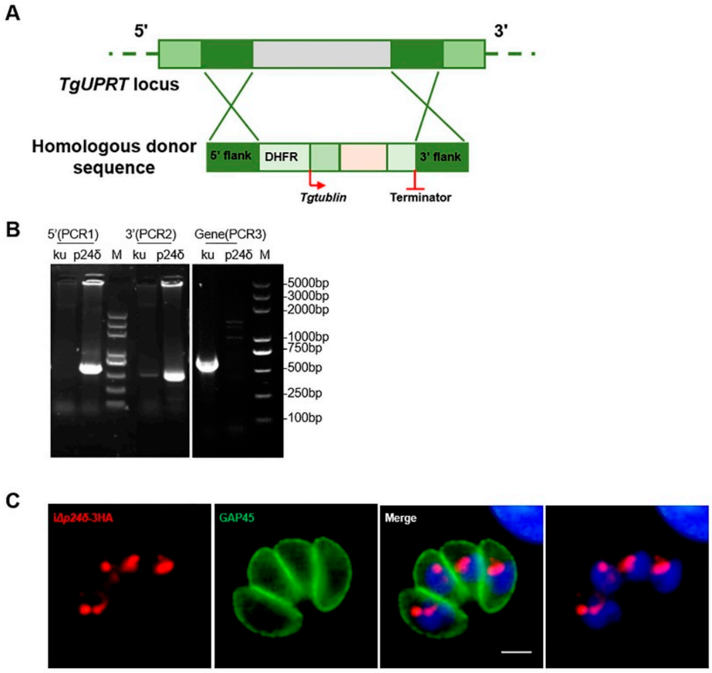

Figure S4. Construction of Tgp24δ complementation in the *UPRT* locus. (A) Schematic diagram illustrating Tgp24 complementation in the *UPRT* locus. (B) PCR1 and PCR2 confirmed proper integration of homologous fragments in the 5′ and 3′ regions, respectively, while PCR3 confirmed the deletion of UPRT coding region. (C) IFA confirmed the correct construction of i*Δp24δ* strain. Scale bars, 2 μm.
